# Dietary Riboflavin Intake and the Risk of Stroke: Insights From NHANES 2007–2018

**DOI:** 10.1002/fsn3.70282

**Published:** 2025-05-15

**Authors:** Haimin Jin, Yaxi Zhang

**Affiliations:** ^1^ Department of Neurology Wenzhou Central Hospital Wenzhou China

**Keywords:** cross‐sectional study, intake, National Health and Nutrition Examination, riboflavin, stroke

## Abstract

There have been few studies examining the connection between dietary riboflavin intake and stroke. We aimed to investigate the prevalence of stroke and the association between dietary riboflavin intake and stroke in the United States population. Participants in this extensive, cross‐sectional study were drawn from the National Health and Nutrition Examination Survey (NHANES) conducted between 2007 and 2018. Questionnaires and 24‐h recall interviews were used to gather information on stroke events and dietary intake. To assess the relationships between riboflavin intake and stroke risk, multivariate logistic regression, restricted cubic spline, and subgroup analysis were used. The incidence of stroke was 3.67% among the 20,776 participants in the research. We discovered that a higher riboflavin consumption was linked to a decreased risk of stroke in people in the United States after controlling for all specified factors. For stroke risk, the odds ratio (OR; 95% confidence interval) for those with the highest riboflavin intake was 0.57 (95% CI: 0.38–0.85) compared to those with the lowest intake. Additionally, an L‐shaped relationship between riboflavin intake and stroke risk was discovered by the study, with an inflection point at roughly 4.95 mg/day. According to our research, the risk of stroke was inversely correlated with a high riboflavin consumption. To validate our findings and investigate the causal links, more research is required.

## Graphical abstract

Our findings showed that dietary intake of riboflavin was inversely related to the risk of stroke. The subgroup analysis suggested that this inverse association was more pronounced in males, those with age between 40 and 59 years those with BMI between 25 and 30 kg/m^2^, those who drank at least 12 times last year, and participants without hypertension, diabetes, and hypercholesterolemia. Furthermore, RCS curves showed a non‐linear inverse relationship between riboflavin intake and stroke risk.

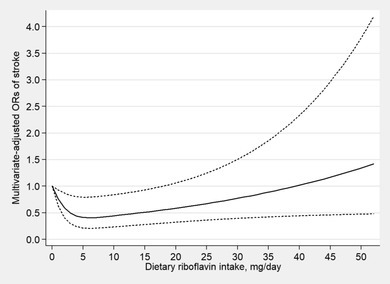

## 1 Introduction

Stroke is a common neurological condition that can originate from structural abnormalities in vascular tissues, which produce parenchymal hemorrhage and subsequent neuronal injury, or from a reduced blood supply to the brain because of occlusion or stenosis (Shehjar et al. 2003). Stroke's high rates of morbidity, death, and disability make it a major public health problem (Feigin et al. 2022). Statistics show that between 1990 and 2019, the prevalence of stroke increased by 85% worldwide, with 1.01 million stroke epidemic cases in 2019 (Roth et al. 2020; GBD 2019 Stroke Collaborators 2021). Stroke claims the lives of over 86.5 out of every 100,000 persons annually (Mai and Liang 2020). Even stroke survivors may experience cognitive, linguistic, and strength impairments (Huang et al. 2018). Additionally, the projected yearly global cost of stroke exceeds $89.1 billion, putting a significant burden on health care systems and having a huge socioeconomic impact (Owolabi et al. 2022). Therefore, reducing the prevalence of stroke requires identifying factors that may be prevented and controlled.

Dietary patterns and stroke have become increasingly linked and have attracted a lot of attention (Teng et al. 2024). Previous research has examined the associations between stroke risk and dietary inflammatory index (Mao et al. 2024), copper (Yang et al. 2022), magnesium (Sun et al. 2023), potassium (Fang et al. 2000), zinc (Huang et al. 2024), vitamin C (Tang et al. 2022), vitamin B6 (Wang et al. 2024), and vitamin B12 (Yahn et al. 2021). Notably, not enough research has been done on the connection between dietary riboflavin (vitamin B2) consumption and stroke, but the benefits of riboflavin in the treatment of stroke have already been reported (Silva‐Araújo et al. 2024; Betz et al. 1994). The human body cannot produce riboflavin on its own (Mohedano et al. 2019). Although certain gut bacteria can synthesize small amounts, this endogenous production is insufficient to meet physiological requirements (Liu et al. 2020). Therefore, adequate riboflavin status must be obtained primarily through a balanced diet or supplements. As a cofactor for methylenetetrahydrofolate reductase (MTHFR), riboflavin deficiency may lead to homocysteine (Hcy) accumulation (Strain et al. 2004; McNulty et al. 2019). Elevated Hcy levels are an independent risk factor for stroke (Strain et al. 2004; McNulty et al. 2019), suggesting that optimizing riboflavin intake may represent a modifiable dietary strategy for stroke risk reduction.

Given that the relationship between dietary riboflavin consumption and stroke risk has not been fully investigated, this study sought to fill the knowledge gaps by examining dietary riboflavin consumption and the dose–response relationship between dietary riboflavin intake and stroke in people in the United States.

## 2 Methods

### 2.1 Study Population

Evaluating the nutritional and health status of non‐institutionalized people in the United States was the main objective of the National Center for Health Statistics' (NCHS) biennial NHANES nationwide program. Its primary objective was to gain a comprehensive understanding of contemporary disease trends and offer data for developing public health policies. All NHANES data was publicly available at no cost at https://www.cdc.gov/nchs/nhanes/index.htm. Participants gave written informed consent, and the NCHS Ethics Review Committee approved the protocol (Qiu et al. 2024).

**FIGURE 1:**
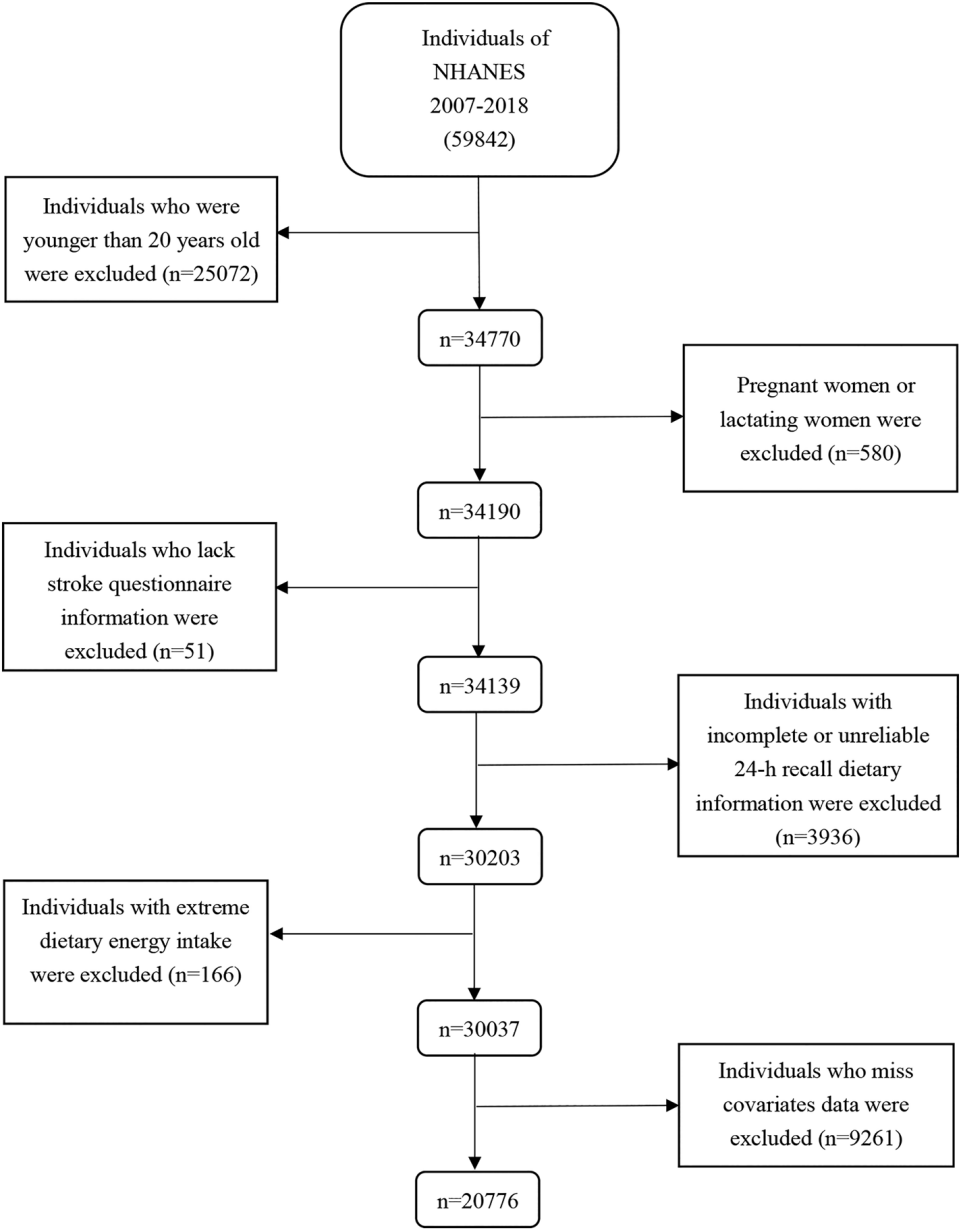
Flow chart of the screening process for the selection of eligible participants. NHANES: National Health and Nutrition Examination Survey.

For this investigation, data was gathered from 59,842 people throughout six successive NHANES cycles (2007–2018). The following particular exclusion criteria were used (1): Age < 20 years (*n* = 25,072); (2): Women who were pregnant or breastfeeding (*n* = 580); (3): Stroke diagnosis not obtained (*n* = 51); (4): Dietary information not received (*n* = 3936); (5): Inappropriate energy intake (*n* = 166); (6): Covariates data missing (*n* = 9261). A total of 20,776 participants were chosen for additional investigation following a thorough data screening process. A thorough flowchart that illustrated the study participant recruitment process is shown in Figure 1 and Table S1.

### 2.2 Assessment of the Dietary Riboflavin Intake

Two 24‐h recall interviews were used to document each participant's nutritional intake for the NHANES dataset. Three to ten days following the first in‐person interview, a telephone interview was conducted. Dietary data was encoded and converted into total nutrient consumption using the United States Department of Agriculture (USDA) survey nutrient database. The 24‐h dietary supplement usage component was performed following the 24‐h dietary survey for food and beverage consumption. Dietary riboflavin consumption was determined by averaging the intake from two 24‐h meal surveys and two 24‐h supplement surveys. Additionally, dietary riboflavin intake was divided into four groups according to quartile (Quartile 1: < 25th percentile, Quartile 2: 25–50th percentile, Quartile 3: 50–75th percentile and Quartile 4: ≥ 75th percentile) (Wu et al. 2020; Jin et al. 2024), and was divided into two groups according to recommended dietary allowance (RDA, 1.3 mg/day for males and 1.1 mg/day for females) (Dietary guidelines for Americans 2020).

### 2.3 Assessment of Stroke

We categorized stroke outcome based on self‐reported survey replies. In interviews, participants were asked, “Has a doctor or other health professional ever told you that you have had a stroke?” An stroke result would be deemed to have occurred if the respondent selected “Yes” (Qiu et al. 2024).

### 2.4 Covariates

We sought to include a wide variety of factors known to skew stroke outcomes, drawing on prior research and biological considerations. These covariates included age, gender, race, sleeping disorder, education level, poverty‐income ratio (PIR), body mass index (BMI), marital status, work activity, recreational activity, smoking status, drinking status, diabetes, hypertension, hypercholesterolemia, dietary energy intake, dietary fat intake, and serum folate concentration. Table S2 provided a thorough explanation and classification of the covariates.

### 2.5 Statistical Analysis

For the primary statistical analysis in this investigation, we used SPSS version 27.0 and Stata 15.0 (StataCorp, College Station, TX). New sample weights were established by integrating continuous data from six 2‐year cycles in accordance with the NHANES analysis requirements (Wu et al. 2020).

While categorical variables were shown as numbers (*n*) and percentages (%) in the baseline data, continuous variables were represented by means and standard deviations. The variations in individual characteristics between the groups with and without stroke outcomes were then evaluated using the student's *t*‐test and chi‐square test.

Regarding riboflavin intake, the four groups were Quartile 1 (< 1.42 mg/day), Quartile 2 (1.42 to 2.11 mg/day), Quartile 3 (2.11 to 3.26 mg/day), and Quartile 4 (≥ 3.26 mg/day). The logistic regression model was used to investigate the relationships between dietary riboflavin intake and stroke risk. Model 1 was adjusted for age and sex, while Model 2 was adjusted for every variable listed in Table S1. Subgroup and interaction analyses were used to assess the results' stability. We used the restricted cubic splines (RCS) model to address the non‐linear correlations between response variables and continuous variables. When the two‐sided *p* value was less than 0.05, the study's conclusions were deemed statistically significant.

## 3 Results

### 3.1 Characteristics of the Study Population

A total of 59,842 NHANES participants from 2007 to 2018 were first included in the current analysis. The inclusion and exclusion criteria finally resulted in the enrollment of 20,776 people with food recall data. The average age of all research participants was 49.67 years, and slightly less than half (49.04%) of the group was male. 763 of them (3.67%) had previously experienced a stroke. Individuals with a history of stroke also had higher odds of having diabetes (39.19% vs. 16.95%), hypertension (86.37% vs. 53.37%), hypercholesterolemia (62.25% vs. 35.65%), and being older (65.66 years vs. 49.06 years). Those who experienced a stroke also had lower dietary riboflavin intakes (3.65 mg/day vs. 3.94 mg/day), with the study population's mean dietary riboflavin consumption being 3.85 mg/day. Comprehensive clinical and demographic data were shown in Table 1.

**TABLE 1:** Characteristics of participants by stroke status, NHANES 2007–2018.

Characteristics	Overall	Stroke	Non‐stroke	*p*
Number of subjects (%)	20,776 (100.00)	763 (100.00)	20,013 (100.00)	
Gender (%)^a^				
Male	10,188 (49.04)	372 (48.75)	9816 (49.05)	0.883
Female	10,588 (50.96)	391 (51.25)	10,197 (50.95)	
Race (%)^a^				
Mexican American	3020 (14.54)	63 (8.26)	2957 (14.78)	< 0.001
Other Hispanic	2066 (9.94)	49 (6.42)	2017 (10.08)	
Non‐Hispanic White	9420 (45.34)	405 (53.08)	9015 (45.05)	
Non‐Hispanic Black	4168 (20.06)	197 (25.82)	3971 (19.84)	
Other Race	2102 (10.12)	49 (6.42)	2053 (10.26)	
Age (%)^a^				
20–39	6799 (32.73)	34 (4.46)	6765 (33.80)	< 0.001
40–59	7026 (33.82)	173 (22.67)	6853 (34.24)	
≥ 60	6951 (33.46)	556 (72.87)	6395 (31.95)	
Body mass index (%)^a^				
< 25 kg/m^2^	5861 (28.21)	179 (23.46)	5682 (28.39)	0.006
25–30 kg/m^2^	6825 (32.85)	254 (33.29)	6571 (32.83)	
≥ 30 kg/m^2^	8090 (38.94)	330 (43.25)	7760 (38.77)	
Educational level (%)^a^				
< High school	1817 (8.75)	106 (13.89)	1711 (8.55)	< 0.001
High school	7526 (36.22)	356 (46.66)	7170 (35.83)	
> High school	11,433 (55.03)	301 (39.45)	11,132 (55.62)	
Smoking status (%)^a^				
Yes	5203 (25.04)	282 (36.96)	4921 (24.59)	< 0.001
No	15,573 (74.96)	481 (63.04)	15,092 (75.41)	
Drinking status (%)^a^				
Yes	14,094 (67.84)	478 (62.65)	13,616 (68.04)	0.002
No	6682 (32.16)	285 (37.35)	6397 (31.96)	
Poverty‐income ratio (%)^a^				
< 1.00	4274 (20.57)	185 (24.25)	4089 (20.43)	0.011
≥ 1.00	16,502 (79.43)	578 (75.75)	15,924 (79.57)	
Recreational activity (%)^a^				
Vigorous	4692 (22.58)	35 (4.59)	4657 (23.27)	< 0.001
Moderate	5543 (26.68)	179 (23.46)	5364 (26.80)	
Other	10,541 (50.74)	549 (71.95)	9992 (49.93)	
Work activity (%)^a^				
Vigorous	4204 (20.23)	100 (13.11)	4104 (20.51)	< 0.001
Moderate	4571 (22.00)	141 (18.48)	4430 (22.14)	
Other	12,001 (57.76)	522 (68.41)	11,479 (57.36)	
Sleeping disorder (%)^a^				
Yes	656 (3.16)	127 (16.64)	529 (2.64)	< 0.001
No	20,120 (96.84)	636 (83.36)	19,484 (97.36)	
Marital status (%)^a^				
Married/living with partner	12,452 (59.93)	403 (52.82)	12,049 (60.21)	< 0.001
Widowed/divorced/separated	4572 (22.01)	303 (39.71)	4269 (21.33)	
Never married	3752 (18.06)	57 (7.47)	3695 (18.46)	
Diabetes status (%)^a^				
Yes	3692 (17.77)	299 (39.19)	3393 (16.95)	< 0.001
No	17,084 (82.23)	464 (60.81)	16,620 (83.05)	
Hypertension status (%)				
Yes	11,339 (54.58)	659 (86.37)	10,680 (53.37)	< 0.001
No	9437 (45.42)	104 (13.63)	9333 (46.63)	
Hypercholesterolemia status (%)^a^				
Yes	7610 (36.63)	475 (62.25)	7135 (35.65)	< 0.001
No	13,166 (63.37)	288 (37.75)	12,878 (64.35)	
Dietary riboflavin intake (mg/day)^b^	3.85 ± 9.74	3.65 ± 9.66	3.94 ± 11.64	0.039
Dietary fat intake (g/day)^b^	77.91 ± 37.95	70.34 ± 37.35	78.19 ± 37.94	< 0.001
Total energy intake (kcal/day)^b^	2047.76 ± 815.73	1806.69 ± 782.25	2056.95 ± 815.58	< 0.001
Serum folate concentration (nmol/L)^b^	43.18 ± 32.17	41.39 ± 61.63	43.59 ± 30.42	< 0.001

Abbreviation: NHANES, National Health and Nutrition Examination Survey.

^a^ Chi‐square test was used to compare the percentage between participants with and without stroke.

^b^ Student's *t*‐test was used to compare the mean values between participants with and without stroke.

### 3.2 Multivariate Regression Analyses

Table 2 displays the findings of the logistic regression study that examined the relationship between dietary riboflavin intake and stroke risk according to quartiles of intake. In both the basic model and Model 1, a higher dietary riboflavin intake was associated with a decreased risk of stroke; those who consumed more riboflavin (Quartile 4) had a 41% and 56% lower risk of stroke, respectively, than those who consumed less (Quartile 1). Comparing Quartile 4 to Quartile 1, the corresponding odds ratios (ORs) with 95% confidence intervals (CIs) were 0.59 (0.43–0.79) and 0.44 (0.33–0.61) in the crude model and Model 1. The association between riboflavin intake and stroke risk remained negative in Model 2. Compared to Quartile 1, the ORs (95% CIs) for stroke risk were 0.52 (0.35–0.77), 0.56 (0.39–0.81), and 0.57 (0.38–0.85) for those who ingested riboflavin through their diet in Quartiles 2, 3, and 4.

**TABLE 2:** Weighted ORs (95% CIs) for stroke according to quartiles of dietary riboflavin intake, NHANES 2007–2018.

Intake cutoff		Cases/participants^a^	Crude^b^	Model 1^b^	Model 2^b^
			OR (95% CI)	OR (95% CI)	OR (95% CI)
Dietary riboflavin intake (mg/day)					
Quartile 1 (low)	< 1.42	225/5198	1 (ref)	1 (ref)	1 (ref)
Quartile 2	1.42–2.11	163/5192	0.50 (0.36–0.71)**	0.47 (0.32–0.68)**	0.52 (0.35–0.77)**
Quartile 3	2.11–3.26	196/5192	0.53 (0.39–0.72)**	0.47 (0.34–0.65)**	0.56 (0.39–0.81)**
Quartile 4 (high)	≥ 3.26	179/5194	0.59 (0.43–0.79)**	0.44 (0.33–0.61)**	0.57 (0.38–0.85)**
*p*_trend_			< 0.001	< 0.001	< 0.001

*Note:* Model 1 adjusted for age and gender. Model 2 adjusted for gender, body mass index, race, age, smoking status, educational level, marital status, poverty‐income ratio, sleeping disorder, drinking status, hypertension status, recreational activity, work activity, hypercholesterolemia status, diabetes status, dietary energy intake, dietary fat intake and serum folate concentration. Results were dietary‐weighted.

Abbreviations: CI, confidence interval; NHANES, National Health and Nutrition Examination Survey; OR, odd ratio.

^a^ Cases of stroke/number of participants in quartiles.

^b^ Calculated using binary logistic regression.

** *p* < 0.01.

The associations between the dietary riboflavin intake categorized based on RDA and stroke risk were presented in Table 3. In Model 2, compared to people whose dietary riboflavin intakes were below the RDA, those with intake meeting the RDA had a lower risk of stroke, with OR (95% CI) of 0.67 (0.49–0.91).

**TABLE 3:** Weighted ORs (95% CIs) for stroke according to RDA of dietary riboflavin intake, NHANES 2007–2018.

Intake cutoff		Cases/participants^a^	Crude^b^	Model 1^b^	Model 2^b^
			OR (95% CI)	OR (95% CI)	OR (95% CI)
Dietary riboflavin intake (mg/day)					
Below the RDA	< 1.1 for females and < 1.3 for males	144/3374	1 (ref)	1 (ref)	1 (ref)
Met the RDA	≥ 1.1 for females and ≥ 1.3 for males	619/17402	0.64 (0.51–0.82)**	0.51 (0.39–0.67)**	0.67 (0.49–0.91)*

*Note:* Model 1 adjusted for age and gender. Model 2 adjusted for gender, body mass index, race, age, smoking status, educational level, marital status, poverty‐income ratio, sleeping disorder, drinking status, hypertension status, recreational activity, work activity, hypercholesterolemia status, diabetes status, dietary energy intake, dietary fat intake and serum folate concentration. Results were dietary‐weighted.

Abbreviations: CI, confidence interval; NHANES, National Health and Nutrition Examination Survey; OR, odd ratio; RDA, recommended dietary allowance.

^a^ Cases of stroke/number of participants in quartiles.

^b^ Calculated using binary logistic regression.

* *p* < 0.05.

** *p* < 0.01.

### 3.3 Subgroup Analyses

**FIGURE 2:**
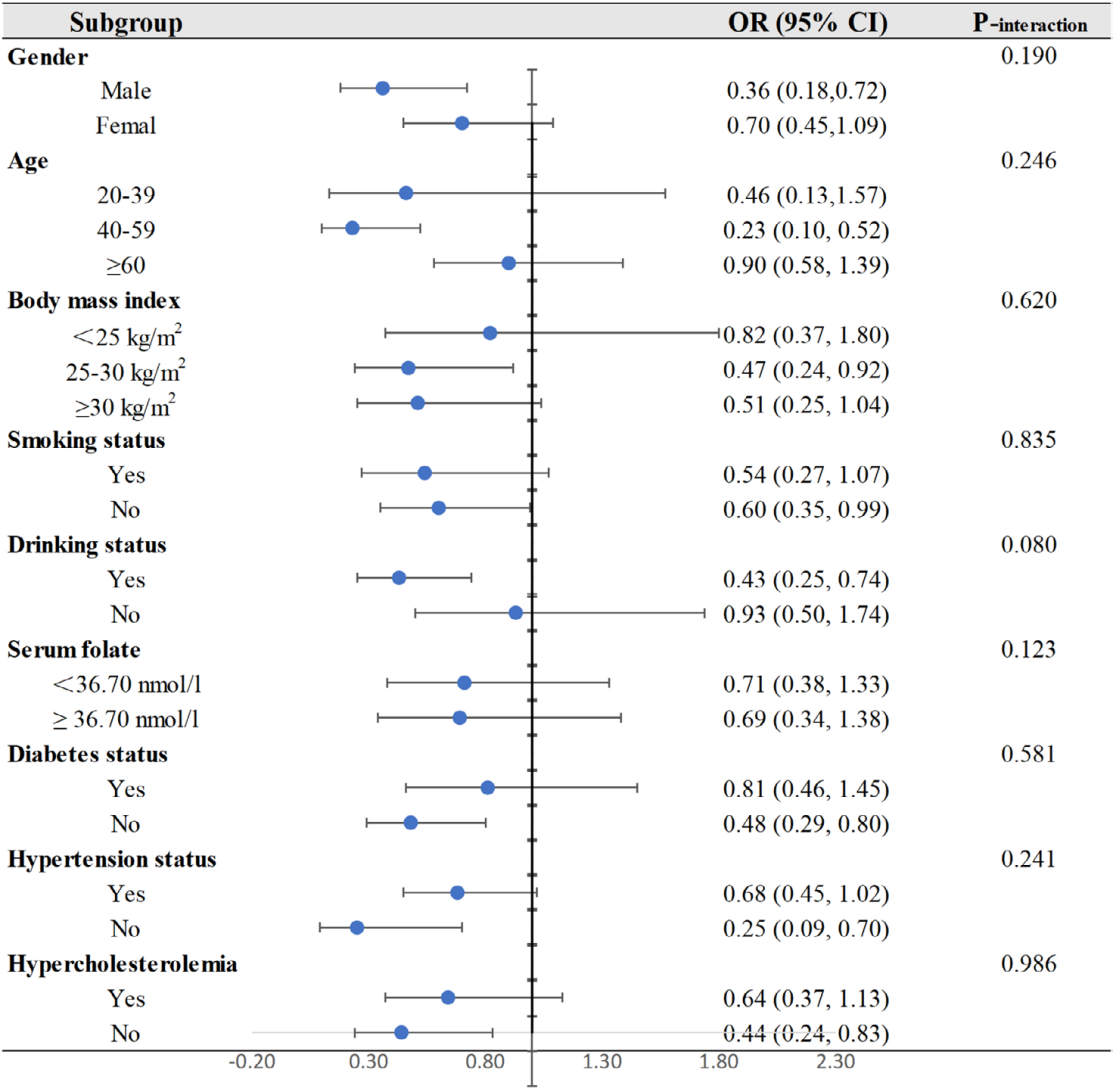
Subgroup analyses for the relationship between dietary riboflavin intake and stroke. CI, confidence interval; OR, odd ratio.

The findings of subgroup analyses and interactions were displayed in a forest plot in Figure 2. Those aged 40–59 years those with a BMI between 25 and 30 kg/m^2^, those who drank at least 12 times last year, those being males, and those who did not have hypertension, diabetes, or hypercholesterolemia were more likely to have an inverse relationship between riboflavin and stroke. Interaction studies revealed that the preventive effect of dietary riboflavin against stroke was unaffected by gender, age, BMI, smoking or drinking status, blood folate concentration, diabetes, hypertension, or hyperlipidemia status.

### 3.4 Dose–Response Analyses

**FIGURE 3:**
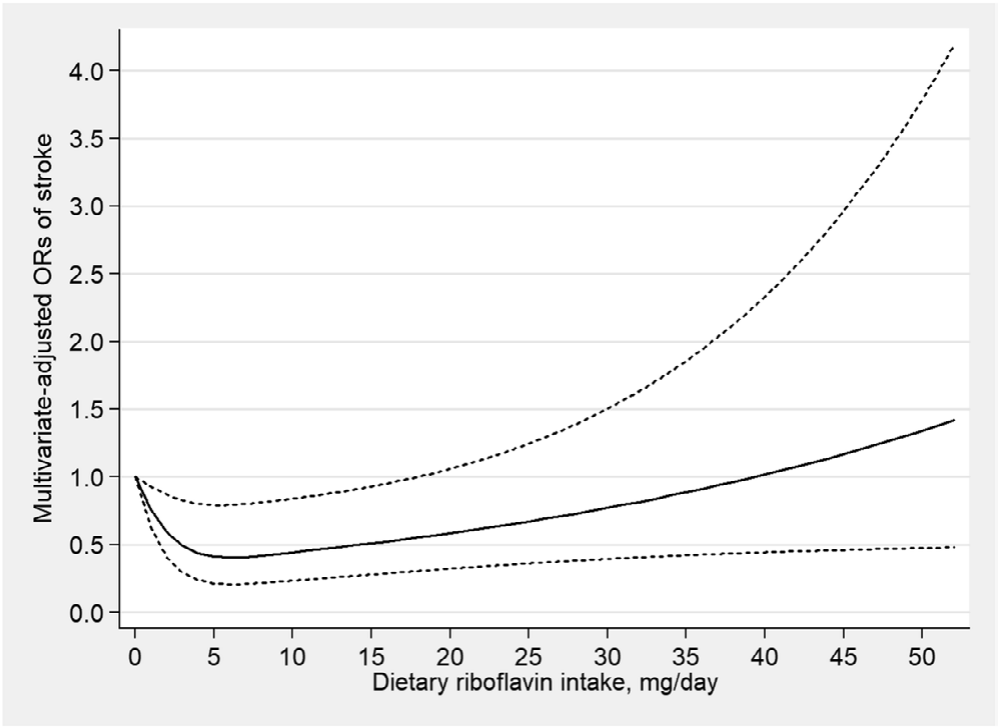
Restricted cubic spline model of the odds ratios of stroke with dietary riboflavin intake. The dashed lines represent the 95% confidence intervals.

A RCS function with three knots was used in a fully adjusted logistic regression model to examine the relationship between dietary riboflavin intake and stroke risk. The results indicated an L‐shaped trend (*p* = 0.007 for nonlinearity) of dietary riboflavin intake with the stroke risk, depicted in Figure 3. An inflection point was identified at 4.95 mg/day in the threshold effect of dietary riboflavin intake on stroke risk.

## 4 Discussion

To the best of our knowledge, this was the first large cross‐sectional study to look at the link between stroke and dietary riboflavin intake. The prevalence of stroke was 3.67% in this study. Our findings showed that dietary intake of riboflavin was inversely related to the risk of stroke. The subgroup analysis suggested that this inverse association was more pronounced in males, those with ages between 40 and 59 years old, those with BMI between 25 and 30 kg/m^2^, those who drank at least 12 times last year, and participants without hypertension, diabetes, and hypercholesterolemia. Furthermore, RCS curves showed a non‐linear inverse relationship between riboflavin intake and stroke risk.

Elevated plasma Hcy levels are a well‐established risk factor for stroke, with studies showing that a 25% reduction in Hcy corresponds to a 19% decrease in stroke risk (Homocysteine Studies Collaboration 2002). Riboflavin plays a critical role in Hcy metabolism as an essential component of the coenzymes flavin adenine dinucleotide (FAD) and flavin mononucleotide (FMN) (Bjørklund et al. 2022; Zaric et al. 2019). Specifically, FAD serves as a cofactor for methylenetetrahydrofolate reductase (MTHFR), which catalyzes the conversion of 5,10‐methylenetetrahydrofolate to 5‐methyltetrahydrofolate—a key methyl donor in the remethylation of Hcy to methionine (Selhub and Miller 1992; McNulty et al. 2012). Meanwhile, FMN is required for generating pyridoxal phosphate (the active form of vitamin B6), which supports the trans‐sulfuration pathway via cystathionine β‐synthase (Strain et al. 2004). Beyond Hcy regulation, riboflavin deficiency may increase stroke risk by impairing myelin synthesis, potentially leading to demyelination (Plantone et al. 2021). Additionally, in the situation of ischemia/anoxia, riboflavin exhibits neuroprotective effects by mitigating oxidative stress, reducing inflammation, and promoting neural repair (Silva‐Araújo et al. 2024).

In the Hcy metabolic pathway, riboflavin is frequently disregarded, yet it is crucial for individuals whose MTHFR gene contains the 677C → T variant (McNulty et al. 2006). The MTHFR 677TT genotype has an average frequency of 12% worldwide (Brattström et al. 1998). The MTHFR gene with the 677C → T variant is more likely to break into monomers and lose the FAD prosthetic group during dilution, which lowers enzyme activity when compared to the MTHFR 677CC genotype (Yamada et al. 2001). The MTHFR enzyme activity in TT cells is roughly 50% that of CC cells when there is sufficient folate and riboflavin present (Strain et al. 2004). The mutant version of the enzyme can be stabilized by a greater riboflavin status, which can either stop the FAD cofactor from leaving the active site or enable its prompt replacement (McNulty et al. 2019; Holmes et al. 2011). The inverse association between dietary riboflavin and stroke may be more significant in individuals with the MTHFR 677TT genotype, but genotype information was not collected in the NHANES database.

Erythrocyte glutathione reductase activation coefficient (EGRac) is regarded as a more reliable biomarker of riboflavin status than dietary intake (McAuley et al. 2016), but this measure is not available in the NHANES database. The correlation between riboflavin intake and EGRac has been discussed in many articles with inconsistent results. Two cross‐sectional studies with a small sample size found no significant association between dietary riboflavin intake and the value of EGRac (Aljaadi et al. 2021; Mataix et al. 2003), whereas multiple intervention trials demonstrated an inverse relationship (Boisvert et al. 1993; Suboticanec et al. 1990; Powers et al. 2011; Bates et al. 1982). Given that the null associations primarily stem from cross‐sectional studies with limited sample sizes—and considering that humans cannot synthesize riboflavin endogenously—we propose that EGRac is indeed responsive to long‐term riboflavin intake but may not reflect short‐term fluctuations in dietary supply. The dose–response analysis revealed an inverse relationship between riboflavin intake and stroke risk, with optimal protection observed at 4.95 mg/day—a level higher than the RDA (1.3 mg/day for adult men; 1.1 mg/day for adult women) (Li et al. 2023). This may be because the RDA is set as the minimum safe level to weigh metabolic needs and prevention of deficiency (Turck et al. 2017), whereas the higher “effective dose” found in the study only targets stroke health benefits.

This study's conclusion that riboflavin was linked to the risk of stroke was in line with other research. In a 1994 study on rat stroke models, Betz and associates demonstrated that riboflavin pretreatment decreased the development of oedema brought on by ischaemia (Betz et al. 1994). When Gariballa and Ullegaddi examined stroke patients in the acute phase right after the infarct, they discovered that a significant percentage of them had poor riboflavin status with the values of EGRac greater than 1.3 (Gariballa 2007). In a clinical trial of 50 stroke patients, those treated with 20 mg of intravenous riboflavin had significantly lower blood glutamate levels at discharge and slightly greater improvement in clinical disability (da Silva‐Candal et al. 2018). A single study reported reduced vitamin B2 levels in stroke patients compared to controls (Ullegaddi et al. 2005).

Our research has a number of advantages. Firstly, using a large, cross‐sectional, and well‐established dataset, we have added reliable evidence to the research of the riboflavin association with stroke. Second, we performed subgroup analyses and interactions, considered and corrected for established possible stroke risk variables, and generated more convincing findings. Third, our study made sure that the data was representative of the country's population by taking into account the intricate sampling design and NHANES weights. Finally, this study provided evidence that appropriate doses of riboflavin intake may be potential strategies for preventing stroke.

Before evaluating the data, a few caveats should be noted. First of all, because this study was cross‐sectional, it would be difficult to fully rule out any confounding variables, which would restrict its capacity to prove causation. Second, even though two 24‐h dietary recalls were used to collect dietary data, recall bias could still exist. Third, there was no riboflavin serum data and MTHFR genotype data in the database. Fourth, there might be a few more confounders that were impossible to completely rule out, such as other elements of a balanced diet and a healthy lifestyle.

## 5 Conclusion

Our findings indicated that dietary intake of riboflavin was inversely related to the risk of stroke, especially in males, those with ages between 40 and 59 years old, those with BMI between 25 and 30 kg/m^2^, those who drank at least 12 times last year, and participants without hypertension, diabetes, and hypercholesterolemia. Given the escalating global stroke burden, these findings suggested riboflavin supplementation might represent a promising preventive strategy for specific at‐risk populations, though further interventional studies were warranted to validate this hypothesis.

## Author Contributions

**Haimin Jin:** conceptualization (equal), data curation (equal), methodology (equal), resources (equal), software (equal), writing – original draft (equal), writing – review and editing (equal). **Yaxi Zhang:** conceptualization (equal), formal analysis (equal), investigation (equal), project administration (equal), supervision (equal), validation (equal), visualization (equal), writing – original draft (equal), writing – review and editing (equal).

## Conflicts of Interest

The authors declare no conflicts of interest.

## Supporting information


**Table S1.** Detailed process of subjects exclusion.

**Table S2.** The classifications of covariates.

## Data Availability

Data of this study can be accessed on the official website of NHANES (https://wwwn.cdc.gov/nchs/nhanes/default.aspx).

## References

[fsn370282-bib-0001] Aljaadi, A. M. , A. M. Wiedeman , S. I. Barr , A. M. Devlin , and T. J. Green . 2021. “Dietary Riboflavin Intake and Riboflavin Status in Young Adult Women Living in Metro Vancouver, Canada.” Current Developments in Nutrition 5, no. 4: nzab021. 10.1093/cdn/nzab021. 33860148 PMC8035065

[fsn370282-bib-0002] Bates, C. J. , A. M. Prentice , M. Watkinson , et al. 1982. “Riboflavin Requirements of Lactating Gambian Women: A Controlled Supplementation Trial.” American Journal of Clinical Nutrition 35, no. 4: 701–709. 10.1093/ajcn/35.4.701. 7072623

[fsn370282-bib-0003] Betz, A. L. , X. D. Ren , S. R. Ennis , and D. E. Hultquist . 1994. “Riboflavin Reduces Edema in Focal Cerebral Ischemia.” Acta Neurochirurgica. Supplementum (Wien) 60: 314–317. 10.1007/978-3-7091-9334-1_84. 7976577

[fsn370282-bib-0004] Bjørklund, G. , M. Peana , M. Dadar , et al. 2022. “The Role of B Vitamins in Stroke Prevention.” Critical Reviews in Food Science and Nutrition 62, no. 20: 5462–5475. 10.1080/10408398.2021.1885341. 33724098

[fsn370282-bib-0005] Boisvert, W. A. , I. Mendoza , C. Castañeda , et al. 1993. “Riboflavin Requirement of Healthy Elderly Humans and Its Relationship to Macronutrient Composition of the Diet.” Journal of Nutrition 123, no. 5: 915–925. 10.1093/jn/123.5.915. 8487103

[fsn370282-bib-0006] Brattström, L. , D. E. Wilcken , J. Ohrvik , and L. Brudin . 1998. “Common Methylenetetrahydrofolate Reductase Gene Mutation Leads to Hyperhomocysteinemia but Not to Vascular Disease: The Result of a Meta‐Analysis.” Circulation 98, no. 23: 2520–2526. 10.1161/01.cir.98.23.2520. 9843457

[fsn370282-bib-0007] da Silva‐Candal, A. , A. Pérez‐Díaz , M. Santamaría , et al. 2018. “Clinical Validation of Blood/Brain Glutamate Grabbing in Acute Ischemic Stroke.” Annals of Neurology 84, no. 2: 260–273. 10.1002/ana.25286. 30014516

[fsn370282-bib-0008] Dietary Guidelines for Americans . 2020. “Dietary Guidelines for Americans. 2015–2020.” https://health.gov/dietaryguidelines/2015/.

[fsn370282-bib-0009] Fang, J. , S. Madhavan , and M. H. Alderman . 2000. “Dietary Potassium Intake and Stroke Mortality.” Stroke 31, no. 7: 1532–1537. 10.1161/01.str.31.7.1532. 10884449

[fsn370282-bib-0010] Feigin, V. L. , M. Brainin , B. Norrving , et al. 2022. “World Stroke Organization (WSO): Global Stroke Fact Sheet 2022.” International Journal of Stroke 17, no. 1: 18–29. 10.1177/17474930211065917. 34986727

[fsn370282-bib-0011] Gariballa, S. 2007. “Ullegaddi R (2007) Riboflavin Status in Acute Ischaemic Stroke.” European Journal of Clinical Nutrition 61, no. 10: 1237–1240. 10.1038/sj.ejcn.1602666. 17299470

[fsn370282-bib-0012] GBD 2019 Stroke Collaborators . 2021. “Global, Regional, and National Burden of Stroke and Its Risk Factors, 1990–2019: A Systematic Analysis for the Global Burden of Disease Study 2019.” Lancet Neurology 20, no. 10: 795–820. 10.1016/S1474-4422(21)00252-0. 34487721 PMC8443449

[fsn370282-bib-0013] Holmes, M. V. , P. Newcombe , J. A. Hubacek , et al. 2011. “Effect Modification by Population Dietary Folate on the Association Between MTHFR Genotype, Homocysteine, and Stroke Risk: A Meta‐Analysis of Genetic Studies and Randomised Trials.” Lancet 378, no. 9791: 584–594. 10.1016/S0140-6736(11)60872-6. 21803414 PMC3156981

[fsn370282-bib-0014] Homocysteine Studies Collaboration . 2002. “Homocysteine and Risk of Ischemic Heart Disease and Stroke: A Meta‐Analysis.” JAMA 288, no. 16: 2015–2022. 10.1001/jama.288.16.2015. 12387654

[fsn370282-bib-0015] Huang, L. , Y. Chen , J. Sun , and L. Xu . 2024. “Exploring the Correlation Between Dietary Zinc Intake and Stroke Risk in Adults Based on NHANES Database.” Neurological Research 46, no. 12: 1113–1121. 10.1080/01616412.2024.2403858. 39510981

[fsn370282-bib-0016] Huang, S. W. , W. C. Chi , K. H. Chang , et al. 2018. “World Health Organization Disability Assessment Schedule 2.0 as an Objective Assessment Tool for Predicting Return to Work After a Stroke.” Disability and Rehabilitation 40, no. 21: 2592–2597. 10.1080/09638288.2017.1342280. 28657351

[fsn370282-bib-0017] Jin, Q. , S. Chen , and X. Ji . 2024. “Associations of Dietary Riboflavin Intake With Coronary Heart Disease in US Adults: A Cross‐Sectional Study of NHANES 2007‐2018.” Frontiers in Nutrition 11: 1467889. 10.3389/fnut.2024.1467889. 39726878 PMC11670662

[fsn370282-bib-0018] Li, H. , J. R. Krall , C. Frankenfeld , and M. Slavin . 2023. “Nutritional Intake of Riboflavin (Vitamin B2) and Migraine: A Cross‐Sectional Analysis of the National Health and Nutrition Examination Survey (NHANES) 2001‐2004.” Nutritional Neuroscience 26, no. 11: 1068–1077. 10.1080/1028415X.2022.2126760. 36175363

[fsn370282-bib-0019] Liu, M. , C. Zhou , Z. Zhang , et al. 2020. “Inverse Association Between Riboflavin Intake and New‐Onset Hypertension: A Nationwide Cohort Study in China.” Hypertension 76, no. 6: 1709–1716. 10.1161/HYPERTENSIONAHA.120.16211. 33131313

[fsn370282-bib-0020] Mai, X. , and X. Liang . 2020. “Risk Factors for Stroke Based on the National Health and Nutrition Examination Survey.” Journal of Nutrition, Health & Aging 24, no. 7: 791–795. 10.1007/s12603-020-1430-4.32744577

[fsn370282-bib-0021] Mao, Y. , J. Weng , Q. Xie , et al. 2024. “Association Between Dietary Inflammatory Index and Stroke in the US Population: Evidence From NHANES 1999–2018.” BMC Public Health 24, no. 1: 50. 10.1186/s12889-023-17556-w. 38166986 PMC10763382

[fsn370282-bib-0022] Mataix, J. , P. Aranda , C. Sánchez , M. A. Montellano , E. Planells , and J. Llopis . 2003. “Assessment of Thiamin (Vitamin B1) and Riboflavin (Vitamin B2) Status in an Adult Mediterranean Population.” British Journal of Nutrition 90, no. 3: 661–666. 10.1079/bjn2003926. 13129473

[fsn370282-bib-0023] McAuley, E. , H. McNulty , C. Hughes , J. J. Strain , and M. Ward . 2016. “Riboflavin Status, MTHFR Genotype and Blood Pressure: Current Evidence and Implications for Personalised Nutrition.” Proceedings of the Nutrition Society 75, no. 3: 405–414. 10.1017/S0029665116000197. 27170501

[fsn370282-bib-0024] McNulty, H. , R. C. Dowey le , J. J. Strain , et al. 2006. “Riboflavin Lowers Homocysteine in Individuals Homozygous for the MTHFR 677C‐>T Polymorphism.” Circulation 113, no. 1: 74–80. 10.1161/CIRCULATIONAHA.105.580332. 16380544

[fsn370282-bib-0025] McNulty, H. , J. J. Strain , K. Pentieva , and M. Ward . 2012. “C(1) Metabolism and CVD Outcomes in Older Adults.” Proceedings of the Nutrition Society 71, no. 2: 213–221. 10.1017/S0029665111003387. 22152927

[fsn370282-bib-0026] McNulty, H. , M. Ward , L. Hoey , C. F. Hughes , and K. Pentieva . 2019. “Addressing Optimal Folate and Related B‐Vitamin Status Through the Lifecycle: Health Impacts and Challenges.” Proceedings of the Nutrition Society 78, no. 3: 449–462. 10.1017/S0029665119000661. 31155015

[fsn370282-bib-0027] Mohedano, M. L. , S. Hernández‐Recio , A. Yépez , et al. 2019. “Real‐Time Detection of Riboflavin Production by *Lactobacillus plantarum* Strains and Tracking of Their Gastrointestinal Survival and Functionality In Vitro and In Vivo Using mCherry Labeling.” Frontiers in Microbiology 10: 1748. 10.3389/fmicb.2019.01748. 31417534 PMC6684964

[fsn370282-bib-0028] Owolabi, M. O. , A. G. Thrift , A. Mahal , et al. 2022. “Primary Stroke Prevention Worldwide: Translating Evidence Into Action.” Lancet Public Health 7, no. 1: e74–e85. 10.1016/S2468-2667(21)00230-9. 34756176 PMC8727355

[fsn370282-bib-0029] Plantone, D. , M. Pardini , and G. Rinaldi . 2021. “Riboflavin in Neurological Diseases: A Narrative Review.” Clinical Drug Investigation 41, no. 6: 513–527. 10.1007/s40261-021-01038-1. 33886098

[fsn370282-bib-0030] Powers, H. J. , M. H. Hill , S. Mushtaq , J. R. Dainty , G. Majsak‐Newman , and E. A. Williams . 2011. “Correcting a Marginal Riboflavin Deficiency Improves Hematologic Status in Young Women in the United Kingdom (RIBOFEM).” American Journal of Clinical Nutrition 93, no. 6: 1274–1284. 10.3945/ajcn.110.008409. 21525198

[fsn370282-bib-0031] Qiu, J. Y. , W. H. Zhang , X. M. Zhu , L. D. Wu , J. H. Huang , and J. Zhang . 2024. “Association Between Dietary Intake of Niacin and Stroke in the US Residents: Evidence From National Health and Nutrition Examination Survey (NHANES) 1999‐2018.” Frontiers in Nutrition 11: 1391023. 10.3389/fnut.2024.1391023. 39101008 PMC11294223

[fsn370282-bib-0032] Roth, G. A. , G. A. Mensah , C. O. Johnson , et al. 2020. “Global Burden of Cardiovascular Diseases and Risk Factors, 1990‐2019: Update From the GBD 2019 Study.” Journal of the American College of Cardiology 76, no. 25: 2982–3021. 10.1016/j.jacc.2020.11.010. 33309175 PMC7755038

[fsn370282-bib-0033] Selhub, J. , and J. W. Miller . 1992. “The Pathogenesis of Homocysteinemia: Interruption of the Coordinate Regulation by S‐Adenosylmethionine of the Remethylation and Transsulfuration of Homocysteine.” American Journal of Clinical Nutrition 55, no. 1: 131–138. 10.1093/ajcn/55.1.131. 1728812

[fsn370282-bib-0034] Shehjar, F. , B. Maktabi , Z. A. Rahman , et al. 2003. “Stroke: Molecular Mechanisms and Therapies: Update on Recent Developments.” Neurochemistry International 162: 105458. 10.1016/j.neuint.2022.105458. PMC9839659 36460240

[fsn370282-bib-0035] Silva‐Araújo, E. R. D. , R. Manhães‐de‐Castro , P. B. Pontes , et al. 2024. “Effects of Riboflavin in the Treatment of Brain Damage Caused by Oxygen Deprivation: An Integrative Systematic Review.” Nutritional Neuroscience 27, no. 9: 989–1007. 10.1080/1028415X.2023.2288387. 38095869

[fsn370282-bib-0036] Strain, J. J. , L. Dowey , M. Ward , K. Pentieva , and H. McNulty . 2004. “B‐Vitamins, Homocysteine Metabolism and CVD.” Proceedings of the Nutrition Society 63, no. 4: 597–603. 10.1079/pns2004390. 15831132

[fsn370282-bib-0037] Suboticanec, K. , A. Stavljenić , W. Schalch , and R. Buzina . 1990. “Effects of Pyridoxine and Riboflavin Supplementation on Physical Fitness in Young Adolescents.” International Journal for Vitamin and Nutrition Research 60, no. 1: 81–88. 2387675

[fsn370282-bib-0038] Sun, P. , Z. Wang , B. Li , and S. Chen . 2023. “Association of Dietary Magnesium Intake With the Risk of Stroke Among Adults.” International Heart Journal 64, no. 6: 1002–1009. 10.1536/ihj.23-299. 37967982

[fsn370282-bib-0039] Tang, X. , H. Liu , Y. Xiao , L. Wu , and P. Shu . 2022. “Vitamin C Intake and Ischemic Stroke.” Frontiers in Nutrition 9: 935991. 10.3389/fnut.2022.935991. 35911106 PMC9330473

[fsn370282-bib-0040] Teng, T. Q. , J. Liu , F. F. Hu , Q. Q. Li , Z. Z. Hu , and Y. Shi . 2024. “Association of Composite Dietary Antioxidant Index With Prevalence of Stroke: Insights From NHANES 1999‐2018.” Frontiers in Immunology 15: 1306059. 10.3389/fimmu.2024.1306059. 38524123 PMC10957548

[fsn370282-bib-0041] Turck, D. , J. L. Bresson , B. Burlingame , et al. 2017. “Dietary Reference Values for Riboflavin.” EFSA Journal 15, no. 8: e04919. 10.2903/j.efsa.2017.4919. 32625611 PMC7010026

[fsn370282-bib-0042] Ullegaddi, R. , H. J. Powers , and S. E. Gariballa . 2005. “Antioxidant Supplementation Enhances Antioxidant Capacity and Mitigates Oxidative Damage Following Acute Ischaemic Stroke.” European Journal of Clinical Nutrition 59, no. 12: 1367–1373. 10.1038/sj.ejcn.1602248. 16091766

[fsn370282-bib-0043] Wang, C. , B. Li , Q. Zhu , et al. 2024. “Dietary Vitamin B6 Intake and Stroke Are Negatively Associated in Adults: A Cross‐Sectional Study From the NHANES.” Heliyon 10, no. 10: e31125. 10.1016/j.heliyon.2024.e31125. 38778939 PMC11109891

[fsn370282-bib-0044] Wu, Y. , S. Li , W. Wang , and D. Zhang . 2020. “Associations of Dietary Vitamin B1, Vitamin B2, Niacin, Vitamin B6, Vitamin B12 and Folate Equivalent Intakes With Metabolic Syndrome.” International Journal of Food Sciences and Nutrition 71, no. 6: 738–749. 10.1080/09637486.2020.1719390. 31986943

[fsn370282-bib-0045] Yahn, G. B. , J. E. Abato , and N. M. Jadavji . 2021. “Role of Vitamin B12 Deficiency in Ischemic Stroke Risk and Outcome.” Neural Regeneration Research 16, no. 3: 470–474. 10.4103/1673-5374.291381. 32985467 PMC7996019

[fsn370282-bib-0046] Yamada, K. , Z. Chen , R. Rozen , and R. G. Matthews . 2001. “Effects of Common Polymorphisms on the Properties of Recombinant Human Methylenetetrahydrofolate Reductase.” Proceedings of the National Academy of Sciences of the United States of America 98, no. 26: 14853–14858. 10.1073/pnas.261469998. 11742092 PMC64948

[fsn370282-bib-0047] Yang, L. , X. Chen , H. Cheng , and L. Zhang . 2022. “Dietary Copper Intake and Risk of Stroke in Adults: A Case‐Control Study Based on National Health and Nutrition Examination Survey 2013–2018.” Nutrients 14, no. 3: 14030409. 10.3390/nu14030409. PMC8839334 35276768

[fsn370282-bib-0048] Zaric, B. L. , M. Obradovic , V. Bajic , M. A. Haidara , M. Jovanovic , and E. R. Isenovic . 2019. “Homocysteine and Hyperhomocysteinaemia.” Current Medicinal Chemistry 26, no. 16: 2948–2961. 10.2174/0929867325666180313105949. 29532755

