# Supplementary material for: Dietary Riboflavin Intake and the Risk of Stroke: Insights From NHANES 2007–2018
Source: Food Sci Nutr. 2025 May 15;13(5):e70282. doi: 10.1002/fsn3.70282 (PMC12079503; doi:10.1002/fsn3.70282)
Supplement: Supplementary file 1 — Table S1. Detailed process of subjects exclusion. [file FSN3-13-e70282-s001.docx]

**Table S1 Detailed process of subjects exclusion**

| Step | The number of subjects before exclusion | The reasons of exclusion | NO.(subjects) | The number of subjects after exclusion |
| --- | --- | --- | --- | --- |
| 1 | 59842 | Age ＜20 | 25072 | 34770 |
| 2 | 34770 | pregnant or lactating women | 580 | 34190 |
| 3 | 34190 | stroke diagnosis not obtained (who lack information on the diagnosis of stroke) | 51 | 34139 |
| 4 | 34139 | dietary information missing (participants with incomplete or unreliable 24-h recall dietary data) | 3936 | 30203 |
| 5 | 30203 | inappropriate energy intake (＜ 500 or [≥](https://baike.baidu.com/item/%E2%89%A5?fromModule=lemma_inlink) 5000 kcal/day for females, and ＜ 500 or [≥](https://baike.baidu.com/item/%E2%89%A5?fromModule=lemma_inlink) 8000 kcal/day for males) | 166 | 30037 |
| 6 | 30037 | covariates data missing | 9261 | 20776 |
